# A standardized construct of blocked threaded wires for treating complex three-part proximal humerus fractures. A long-term follow-up of a previously published series

**DOI:** 10.1016/j.jseint.2025.101437

**Published:** 2025-12-15

**Authors:** Yuri Piccolo, Vittorio Candela, Daniele De Meo, Carmine Zoccali, Stefano Gumina

**Affiliations:** Department of Anatomical, Histological, Forensic Medicine and Orthopaedics Sciences, Sapienza University of Rome, Rome, Italy

**Keywords:** Proximal humerus fractures, Blocked threaded wires, Percutaneous fixation, Percutaneous pinning, Long-term outcomes, Three-part proximal humerus fractures, Plate fixation of humeral fractures

## Abstract

**Background:**

Percutaneous pinning is classically considered an option for treating proximal humerus fractures (PHFs) in elderly low-demanding patients; recently, promising clinical and radiographic medium-term outcomes have been documented after the treatment of displaced PHF using different configurations of blocked threaded wires. However, long-term follow-up (FU) is still lacking. The aim of the present study was to evaluate the clinical and radiographic outcomes of a previously published midterm FU cohort after a minimum of 8 years.

**Methods:**

In this observational study, all 52 patients from the midterm outcome paper were asked to visit our institution for consultation and X-rays. Patients who could not return for an on-site consultation because of poor health completed a self-administered questionnaire with the assistance of his/her general practitioner, and the responses were finalized via a telephone interview. The individual relative Constant-Murley score and the visual analog scale (VAS) were recorded. In radiographic evaluation, both arthritis progression and signs of avascular necrosis were recorded. Complications and reoperation were registered and classified as early (<2 years) and delayed (>2 years).

**Results:**

Of the initial 52 patients, 2 died before the long-term evaluation and 3 were lost to FU. Clinical data were thus obtained for 47 patients (90%), and radiographic data were obtained in 40 patients (77%). The minimum follow-up was 8 years [range: 96-118 months; mean (standard deviation): 102 (4.5) months]. The mean patient age was 68.7 years (standard deviation: 6.3). The mean individual relative Constant-Murley score at the final FU was 83.5%. Regarding the VAS, 36 patients referred their pain as 0 (76.5%), 8 as 1 (17%) and 3 as 2 (6.5%). According to the radiographic assessment, avascular necrosis was present in 2 patients (5%) while 2 patients developed signs of arthritis (Samilson Prieto 2). VAS score was 1/10 in patients with avascular necrosis, whereas a VAS of 2/10 was registered in patients with arthritis. No additional major complications occurred beyond the one previously reported in the midterm analysis: a fracture nonunion who refused any further treatment due to comorbidities. Two superficial infections treated with 5 days of oral antibiotics occurred during the midterm FU. One patient referred an arthroscopic rotator cuff repair 5 years postsurgery with symptoms starting 5 months before the procedure.

**Conclusion:**

Treatment of complex PHF with a construct of blocked threaded wires after an anatomical open/mini-open reduction led to excellent clinical and radiological outcomes with a low rate of complications compared with published results of the other surgical options.

Proximal humerus fractures (PHFs) are the seventh most frequent fractures in adults,^30^ their prevalence varies from 4% to 10%^4^^,^^7^^,^^22^^,^^26^^,^^30^ of all fractures and in patients older than 65 years and only wrist and femoral neck fractures are more frequent.^24^

Percutaneous pinning is classically considered an option for treating PHF only in elderly low demanding patients; in recent years, promising clinical and radiographic medium-term outcomes have been documented after the treatment of displaced PHF using different configurations of blocked threaded wires (BTWs).^5^^,^^8^^,^^12^^,^^18^ Despite the increasing biomechanical and clinical interest in BTW,^9^ the long-term clinical durability, maintenance of reduction over time, and the profile of late complications remain inadequately documented in the literature.

In 2019, the results of a consecutive series of patients with complex PHF treated with a novel construct of BTWs were published,^18^ demonstrating excellent clinical and radiological outcomes, comparable to those documented by locking plate users^1^^,^^3^^,^^11^^,^^13^^,^^14^^,^^16^^,^^23^^,^^25^^,^^27^, ^28^, ^29^ but with a dramatically inferior rate of complications, after a midterm follow-up. The aim of the present study was to evaluate the clinical and radiographic outcomes of the same cohort after a minimum follow-up (FU) of 8 years and to document rates of complications and reoperations.

## Materials and methods

The original paper included a consecutive series of 52 patients with a mean follow-up of 22 months (range, 14-28 months), treated between January 2015 and December 2016 at our Institution for complex three-part PHF classified according to Hertel Lego.^18^^,^^21^

All patients received osteosynthesis after a percutaneous approach or a mini-open approach, with a 3 cm skin incision, reduction. Four pairs of BTWs were percutaneously inserted and connected to an external locking frame with 1 carbon fiber bar that stabilized the construct, according to the technique previously published.^18^ No periosteal stripping or evacuation of the fracture hematoma was performed. Then the shoulder was immobilized in a sling at 15° of abduction for 30 days postoperative. The patients were instructed to begin exercises of the wrist, hand, and fingers, as well as the flexion and extension movements of the elbow, starting from the first postoperative day. The pin sites were disinfected every week and the external frame was routinely removed after 45 days.

All patients signed an informed consent according to the declaration of Helsinki and agreed to participate in the study.

All 52 patients from the medium-term outcome paper were asked to visit our institution for a consultation and X-Rays. Seven patients (13%) who were unable to return for an on-site consultation because of poor health, completed a self-administered questionnaire with the assistance of his/her general practitioner for the clinical evaluation and score recording (individual relative Constant-Murley score [irCS], visual analog scale [VAS]), and the responses were finalized via a telephone interview with the authors. When a patient had died during the follow-up period, his/her general practitioner was asked for the date of death along with data on any complications or revisions. During the FU 2 patients died before the long-term evaluation and 3 patients were lost to follow-up. The irCS^10^ and the VAS were recorded. According to the radiographic evaluation, both arthritis progression (Samilson Prieto and Hamada classifications^19^^,^^31^) and signs of avascular necrosis were recorded. Difference in reduction, considered as a change in the neck shaft angle, between the midterm and final FU, according to the Bahr's criteria^2^ were also documented. Statistical analysis was performed using Fisher's exact test to compare categorical variables between the midterm and long-term follow-up groups. A *P* value < .05 was considered significant. Late complications and reoperation were registered.

## Results

Of the 52 patients of the original paper,^18^ long-term clinical data were obtained for 47 patients (90%). Both clinical and radiographical data were obtained in 40 patients (77%). The minimum follow-up was 8 years [range: 96-118 months; mean (standard deviation): 102 (4.5) months]. The mean patient age was 68.7 years (standard deviation: 6.3).

### Clinical and radiographical evaluation

**Figure 1:**
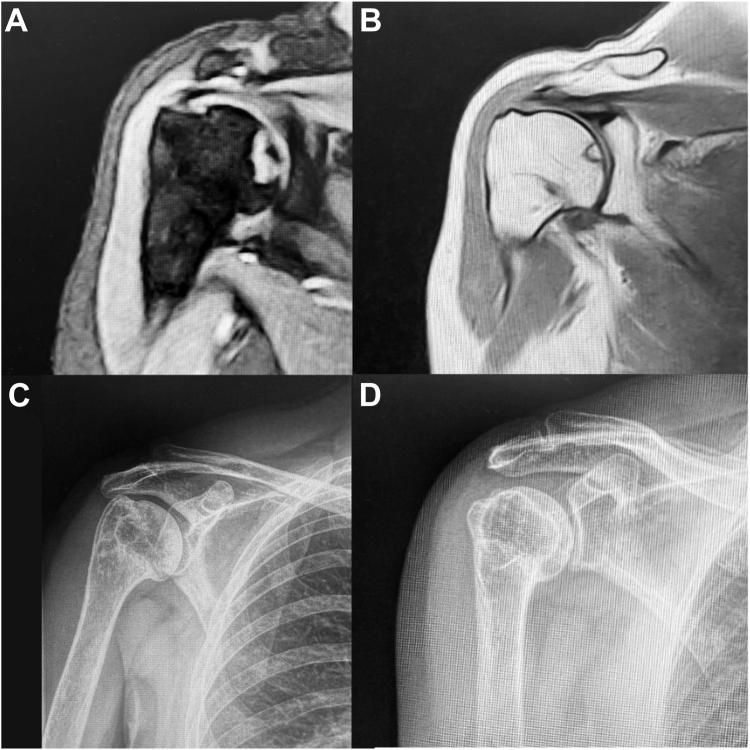
Right shoulder. Final MRI (**A** and **B**) and X-Rays (**C** and **D**) 8.5 years FU. A focal area of avascular necrosis without collapse is present. *FU*, follow-up; *MRI*, magnetic resonance imaging.

The mean irCS at the final FU was 83.5%. Regarding the VAS, 36 patients referred their pain as 0 (76.5%), 8 patients as 1 (17%) and 3 as 2 (6.5%). According to the radiographic assessment (on 40 patients), avascular necrosis was present in 2 patients (5%) (Fig. 1); in both cases subchondral sclerosis with no collapse was present (appeared 16 and 18 months after surgery) and at the final FU a VAS of 1/10 in both patients and a irCS of 82% and 83% were registered.

Two patients developed during the long-term FU radiographic signs of arthritis (both Samilson Prieto 2). In both these 2 patients, VAS was registered as 2/10 and irCS was 78% and 79%.

**Figure 2:**
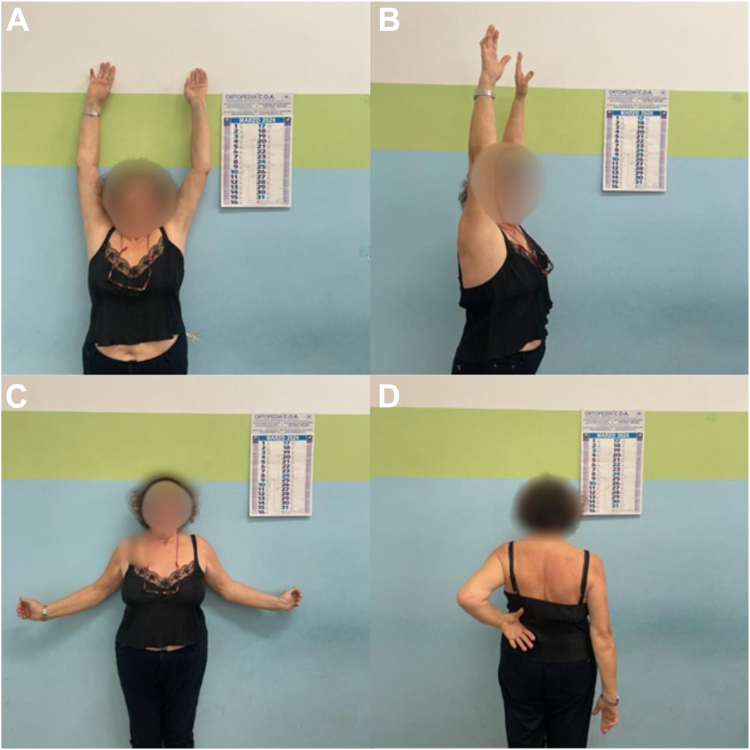
Final clinical FU after 9 years postoperative of the fracture in Figure 3. *FU*, follow-up.

**Figure 3:**
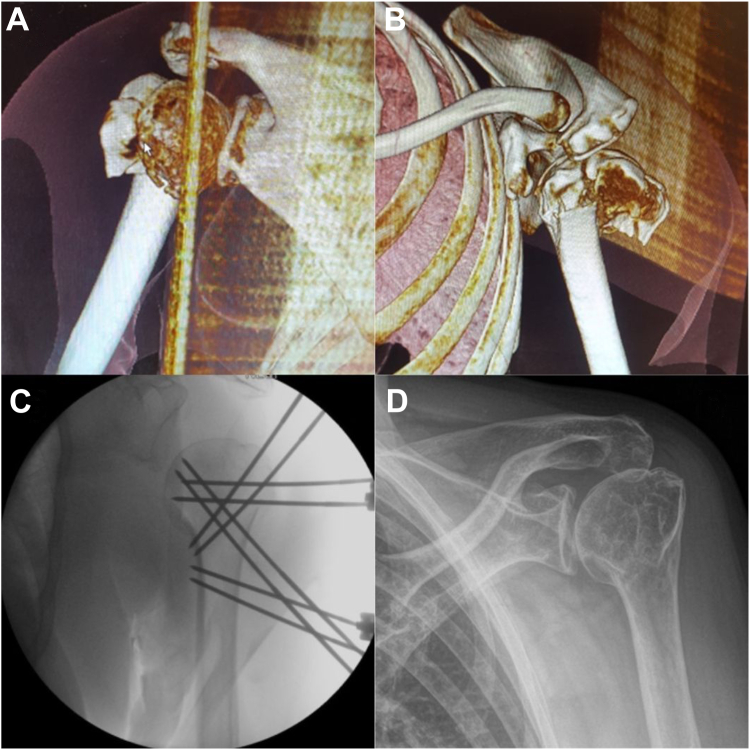
3D CT scan showing a left-sided complex Hertel type 7 proximal humerus fracture (**A** and **B**); intraoperative reduction and definitive fixation with 4 pairs of blocked threaded wires (**C**); final follow-up X-ray 9 years postoperatively (**D**). *3D*, three-dimensional; *CT*, computed tomography.

No statistically significant difference (Table 1) was found according to the midterm FU and the final FU regarding the mean irCS (89.5% vs. 83.5%, *P* value .374) (Fig. 2) and the reduction maintenance, relative to Bahr's criteria (89% vs. 85%, *P* value .757) (Fig. 3).

**Table 1:** Comparison of irCS and Bahr's criteria reduction maintenance between midterm and long-term FU groups.

	Midterm follow-up group	Long-term follow-up group	*P* value
Mean irCS	89.5%	83.5%	.374
Maintenance of reduction according to Bahr's criteria	89%	85%	.757

*FU*, follow-up; *irCS*, individual relative Constant-Murley score.

Values are expressed as percentages.

The only major complication was registered in the midterm period: a fracture nonunion who refused any further treatment due to severe medical conditions. Two superficial infections treated with 5 days of oral antibiotics occurred during the midterm FU.

No delayed complications were identified at long-term FU; no further surgery related to the index procedure in the study cohort was registered at the final FU. One patient referred an arthroscopic rotator cuff repair 5 years after the initial surgery with symptoms relative to rotator cuff tear starting 5 months before the arthroscopic procedure.

## Discussion

The present article investigated both long-term clinical and radiological outcomes and complication rate of a cohort of patients with complex three-part PHFs treated with a system of BTWs in a precise configuration we introduced. The study demonstrated that the use of BTWs after performing an anatomical fracture reduction is associated with excellent clinical outcomes, comparable to those reported when treating those fracture with the gold standard (locking plate), but with a significantly reduced complication rate after a minimum follow-up of 7 years.^1^^,^^3^^,^^11^^,^^13^^,^^14^^,^^16^^,^^23^^,^^25^^,^^27^, ^28^, ^29^

In a previous work, we published the results of the same series but after an average follow-up of 2 years. No differences were found regarding the clinical and radiological outcomes between the midterm and long-term follow-up. Furthermore, no major complications occurred during the long-term FU.

**Figure 4:**
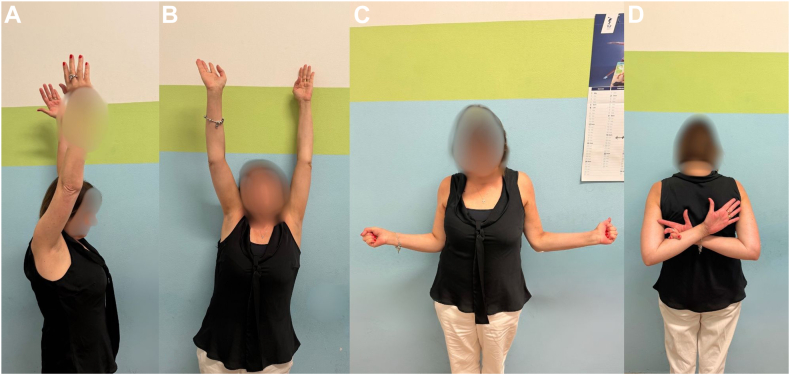
Final clinical 8.5 years FU of the patient with the focal area of avascular necrosis in the right humeral head (as described in Fig. 1). *FU*, follow-up.

In our series, only 2 cases of avascular necrosis (both subchondral sclerosis with no collapse) were documented; patients had a good clinical outcome (Fig. 4) and did not need any further treatment. In fact, as previously demonstrated, avascular necrosis without partial collapse^15^ and without screws^6^ is well tolerated by patients (the system is removed 45 days after surgery). An unquestionable advantage to consider, although not observed in the present series, is the ease of performing an arthroplasty in the event of necrosis associated with collapse. The procedure is technically comparable to primary surgery, as the surgical scar was along the standard deltopectoral approach, deep tissue fibrosis was absent, and there were no in situ fixation device that could prolong surgical time or even lead the surgeon to a two-stage procedure with clinical and psychological consequences for the patient and an additional socioeconomic burden, a consideration particularly relevant when compared to an implant well tolerated by patients during the maintenance period (authors do not recommend its use in individuals with behavioral disorders).

A major complication was recorded in the medium-term follow-up: a case of nonunion in a fracture with comminution of the lateral metaphyseal cortical wall that would have required secondary treatment, which the patient refused due to subsequent medical conditions. In light of the published biomechanical studies,^9^^,^^17^ this complication was predictable; although the BTW system demonstrated the most stable construct for the resistance to bending and torsional forces and also for the transmission of maximum velocity waves,^9^ it was found not to ensure primary stability during the first 45 days in cases with comminution of the lateral wall.^17^ Thanks to these data, we no longer use this system for this type of fracture or extend the maintenance period to 3 months (considering it as a standard external fixator and not a trans-lesional synthesis). Many hypotheses were postulated for justifying these results: our surgical choice does not involve: (a) periosteal nor (b) fracture hematoma removal; (c) although the humeral head is penetrated by the threaded wires, the number of perforations is limited compared to screw insertion in plate fixation. In addition, (d) the earlier removal of the fixation device could facilitate bone healing. The anatomical reduction of the fracture is achieved through a mini-incision of about 3 cm along the deltopectoral approach, which allows us to introduce the instruments necessary to perform the reduction maneuvers. The fractures were fixed using a system composed of 4 pairs of BTWs in a configuration we introduced and defined as isostatic because it prevents collapse and translation of the humeral head. In order to justify these findings associated with the innovative 8-wire construct, a biomechanical two-dimensional validation finite element analysis and parametric optimization were conducted.^17^ The studied construct was found to be biomechanically valid; it only allowed micromovements and generated acceptable pressure stresses on sensitive areas of the fractured humeral head. Recently, Harbrecht et al^20^ performed a brilliant biomechanical study on 21 frozen proximal humerus specimens focusing on the stability of different fixation options for PHFs. The authors compared the gold standard (plate and screw) with 2 different BTW constructs: one in accordance with the manufacturer's recommended technique (3 pairs of threaded wires) and one with an additional pair of threaded wires according to the recommendations of Gumina et al (4 pairs of threaded wires).^18^ BTW application with 8 threaded wires revealed the least relative motion at the fracture site, without a statistically significant difference compared with locking plates, while the use of BTW application with 6 threaded wires resulted in the lowest stability and the highest rates of displacement.

The present study has limitations that need to be addressed: only complex 3 part PHFs have been considered and it is both a limit but also a strength of the present series; 3 part PHFs are the most frequent pattern,^26^ and their treatment represents the daily routine for a shoulder surgeon; however, on the basis of the conducted biomechanical studies,^9^^,^^17^ further evaluations are ongoing on 4-part PHFs associated with a higher risk of avascular necrosis; the irCS is less suitable for evaluating fracture outcomes than for assessing subacromial pathology,^32^ and further limitations are the descriptive nature of the analysis and the absence of comparative groups.

## Conclusions

This study reiterates that, also at long-term follow-up, the treatment of complex PHFs using a standardized and biomechanically reliable construct of BTWs yields excellent clinical and radiographic outcomes, with a very low rate of complications. Randomized controlled trials will be needed to compare the currently debated gold standard with the present fixation concept.

## Disclaimers

Funding: No funding was disclosed by the authors.

Conflicts of interest: The authors, their immediate families, and any research foundation with which they are affiliated and have not received any financial payments or other benefits from any commercial entity related to the subject of this article.
